# Ground State Robustness as an Evolutionary Design Principle in Signaling Networks

**DOI:** 10.1371/journal.pone.0008001

**Published:** 2009-12-01

**Authors:** Önder Kartal, Oliver Ebenhöh

**Affiliations:** 1 Max Planck Institute of Molecular Plant Physiology, Potsdam-Golm, Germany; 2 Institute of Biochemistry and Biology, University of Potsdam, Potsdam-Golm, Germany; 3 Institute for Complex Systems, University of Aberdeen, Aberdeen, United Kingdom; National University of Ireland Galway, Ireland

## Abstract

The ability of an organism to survive depends on its capability to adapt to external conditions. In addition to metabolic versatility and efficient replication, reliable signal transduction is essential. As signaling systems are under permanent evolutionary pressure one may assume that their structure reflects certain functional properties. However, despite promising theoretical studies in recent years, the selective forces which shape signaling network topologies in general remain unclear. Here, we propose prevention of autoactivation as one possible evolutionary design principle. A generic framework for continuous kinetic models is used to derive topological implications of demanding a dynamically stable ground state in signaling systems. To this end graph theoretical methods are applied. The index of the underlying digraph is shown to be a key topological property which determines the so-called kinetic ground state (or off-state) robustness. The kinetic robustness depends solely on the composition of the subdigraph with the strongly connected components, which comprise all positive feedbacks in the network. The component with the highest index in the feedback family is shown to dominate the kinetic robustness of the whole network, whereas relative size and girth of these motifs are emphasized as important determinants of the component index. Moreover, depending on topological features, the maintenance of robustness differs when networks are faced with structural perturbations. This structural off-state robustness, defined as the average kinetic robustness of a network's neighborhood, turns out to be useful since some structural features are neutral towards kinetic robustness, but show up to be supporting against structural perturbations. Among these are a low connectivity, a high divergence and a low path sum. All results are tested against real signaling networks obtained from databases. The analysis suggests that ground state robustness may serve as a rationale for some structural peculiarities found in intracellular signaling networks.

## Introduction

One of the crucial properties of living systems is the ability to sense, process and react to changes in the environment. The basic biological unit capable of translating external information into an appropriate behavior is the cell, whereas the context of cells establishes responses during evolution which are suitable for certain stimuli. As a common principle, the information flow from outside the cell to internal targets like the DNA or the cytoskeleton is mediated by molecular interactions. Signal transduction pathways can amplify the receptor signal by successive steps of activation or deactivation of downstream components. High-throughput methods provide a compelling abundance of data about compounds and their interaction patterns, however, they also reveal a complexity in molecular networks which challenges our understanding of the relationship between structure, dynamics and function. Even the concept of a signaling pathway has been called into question [1].

In recent years attempts have been made to explain topological properties of molecular networks in the light of evolution. The design is regarded as a molecular phenotype which is shaped by natural selection and reflects a successful adaptation towards certain functional demands [2], [3]. Are functional principles in signaling networks present and can we relate them to topology? Clearly, the function of any open living system is governed by its dynamic properties and can therefore not be entirely determined by its topology alone. However, functional flexibility of a system may be constrained or supported considerably by an appropriate structure. Thus, a proper understanding of function should consider the contribution of both the topology and the parameters characterizing the dynamics on this topology.

It is often stated that biological networks have evolved to allow for a robust performance of their functions [4]–[6]. This is attributed to the fact that living systems are constantly subjected to intrinsic and extrinsic noise. Although some findings indicate that living systems do use noise constructively [7], there is no doubt that in many cases maintenance of functionality over a wide range of conditions relies on mechanisms to buffer noise. To provide rationales for particular mechanisms, one needs to be precise about the behavior which is supposed to be robust and the type of uncertainties considered [4]. As the system function is determined by structural as well as kinetic properties, two ways to achieve robustness in signaling networks can be envisaged: by fine-tuning the parameters of the reactions or by establishing a network design for which the considered function is a robust property. The chemotaxis network design of *E. coli* has been shown to provide a robust response both towards extrinsic (changing attractant concentrations [8], [9]) and intrinsic (gene expression [10]) noise.

In line with these studies, and in contrast to purely statistical approaches (see e.g. [11], [12]), we pursue the view that discerning functional advantages or disadvantages of topological properties is more reliable if the corresponding dynamical process is taken into account. However, because in most dynamical models the structure remains fixed or is varied only slightly, the results cannot be readily generalized. An interesting approach to study how network motifs contribute to the stability of the complete network with respect to changes in parameter perturbations has been given by Prill et al. [13]. There, the authors could show that those motifs exhibiting stability for a wider range of kinetic parameters tend to be overrepresented in several analyzed signaling and gene-regulatory networks. Promising approaches to the expansion of these studies on the basic interplay between topology and dynamics to general signaling network structures include more abstract representations like those used in [14] and [15], because they allow for a systematic evaluation of theoretically possible alternative network designs and thus to arrive at theoretical statements of general validity. For example, Heinrich et al. could analyze the effect of the length of a kinase cascade on signaling time and amplitude [15].

Our aim is to study a specific relationship between structure and function by means of a simple framework of signal transduction introduced in [15] and extended in [16], [17]. The model can describe arbitrary activation patterns and is quite general. This enables us to take a graph theoretical approach and use known mathematical results which will highlight new topological properties in signaling systems. The function we consider is a dynamical property which relates to the input-output behavior of the system. Our rather simple hypothesis is that reliable signaling systems should be active only if an input signal (e.g. a ligand) is present. Apparently, an autoactivation in the *signal-off case* could mimic an input signal and cause a behavior detrimental to the cell or the organism. In other words, owing to the fact that extra- and intracellular noise is always present, spurious activations should be dampened out rather than amplified [15]. We will raise this assumption to a robustness principle and analyze for structural properties which support this type of robustness. We propose that during evolution those network topologies have been selected which to a certain extent support the maintenance of ground state stability and thereby prevent noise propagation through the network.

## Methods

### Dynamical System

Consider a network of molecular species 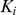 which can exist in a signal transmitting (“active”) and a signal blocking (“inactive”) form. Only in its active form is a species able to activate other molecules. In principle, the adopted framework allows for the description of a large class of signaling systems [15] and more complex processes such as multiple phosphorylations or the effects of scaffold proteins can be included. For simplicity, the analysis is restricted to simple interactions and single activation steps. Assuming mass action kinetics the non-autonomous ordinary differential equations for the concentration of active species, 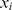, read
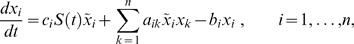
(1)where 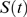 denotes the strength of an external signal, 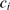 denotes the rate of activation of the 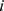-th molecular species by the signal, and the concentration of the inactive form is denoted 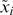. The rate constants 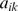 specify the rate of activation of 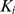 by 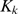. The zero-nonzero structure of the matrix 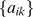 reflects the circuitry of the elements in the signaling network. The constants 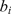 characterize the rate of constitutive deactivation of the species 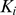, for example by dephosphorylation.

Assuming that production and degradation of each species in the network is always balanced, or a time scale is considered for which changes in total levels are negligible, the conservation relation 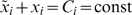 can be used to uncouple the dynamics of inactive and active forms. Furthermore, because the focus is on the impact of signaling topology and the dynamics in the signal-off case, we set 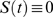 and drastically reduce the kinetic degrees of freedom by imposing 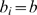 for all 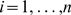. With this we can rescale time (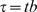) and concentrations (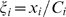) resulting in the corresponding dimensionless and autonomous system
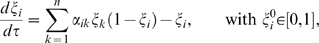
(2)where 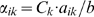.

Due to nonlinearity, multiple stationary states are possible in the system (2). The *off-state* 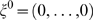 is always a solution characteristic for signal absence. Any stationary state where the concentrations of some or all active species, 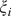, are non-zero is referred to as an *autoactived state* 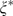 of (2). A bifurcation of the dynamical system occurs if some stationary state looses its stability. We are specifically interested in transcritical bifurcations where at some point in the parameter space stability switches from one state, which is defined as the *ground state*, to an autoactivated state. For the sake of simplicity the off-state is assumed to be the ground state of the signaling system.

### Graph Theory and Algorithms

In terms of graph theory, the quadratic system's matrix 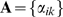 is the *adjacency matrix* of the associated directed graph (or *digraph*) 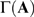. The non-zero entries define the *edges*, i. e. the positive regulatory pattern in the network, whereas the *nodes* symbolize the species. The digraph 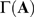 is weighted because the non-zero entries can deviate from unity.

The number of incoming edges of a node is its *in-degree* and accordingly the *out-degree* is defined as the number of outgoing edges. A *source* is a node with in-degree zero, a *sink* has out-degree zero. A series of unidirectional edges is generally called a directed walk or a *path* if and only if each node is visited only once.

The total number of nodes 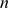 is denoted *order* of the graph and the number of edges 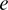 specifies its *size*. If the graph is connected, which is a reasonable assumption for signaling networks, a useful measure is the *connectivity*, 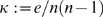. For loop-less graphs (without edges originating and terminating at the same node), it varies in the range of 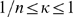 and characterizes the average unspecificity of species in the network.

Any digraph formed by subsets of the original nodes and edges is a *subdigraph*. A spanning subdigraph has the same node set as the original digraph. A subdigraph in which there exists a path between any two nodes is called a *strongly connected component* (SCC). A *cycle* motif is thus a special type of SCC with in-degree and out-degree one for every node. As a closed path it constitutes a positive feedback in the signaling system. An 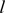-cycle is defined as a cycle of length 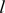, where 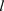 is the number of nodes or edges in the cycle.

Spectral graph theory is concerned with the eigenvalues of a graph. The ordinary spectrum of a graph is the spectrum of the corresponding adjacency matrix. The adjacency matrices of isospectral graphs have the same principal eigenvalue which is real and in terms of graph theory is denoted the *index*, 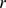, of the graph [18]. Isospectral graphs with distinct topologies are said to belong to the same *index class*.

All calculations were carried out in Matlab (R2008b, The MathWorks Inc., MI, USA), using additional functions from the Boost Graph Library precompiled in MatlabBGL [19]. The paths in the networks were calculated via the random acyclic subdigraph method [20], [21].

## Results

First, we examine how the system structure and parameters determine off-state stability. We show that only the subdigraphs which comprise cycles have an influence in this respect. To analyze how topological characteristics constrain the stable regime a system reduced in kinetic degrees of freedom is considered. A straightforward quantification of kinetic off-state robustness of different motifs is possible in this case. Second, we take into account structural perturbations to discriminate topological features within index classes which are neutral towards kinetic robustness. Three properties which support structural off-state robustness are highlighted. Finally, we analyze signaling networks retrieved from databases for robustness and topological features suggested by our results.

### The Digraph Index Determines Off-State Stability

The Jacobian of (2) evaluated at the off-state reads
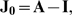
(3)where 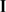 is the identity matrix. It can be shown that the off-state Jacobian has a similar form for Michaelis-Menten type activation kinetics. We can apply Schur's unitary triangularization theorem to (3) and, subsequently, the Perron-Frobenius theorem for non-negative matrices [22] in order to show that the spectral radius of 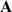 exactly determines the eigenvalue with maximal real part. Let 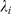 be the eigenvalues of the off-state Jacobian and 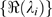 the set comprising the corresponding real parts then
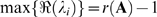
(4)holds. Therefore, a necessary and sufficient condition for off-state stability in the signal-off case is that the index of the signaling network fulfills 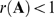. This holds true irrespective of the exact model topology and the precise values of the kinetic parameters in (2). The bifurcation occurs at 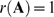.

### The Index Depends on the Feedback Family

With result (4) it is possible to discuss which topological features of the network affect the relevant qualitative change in the dynamic behavior via the digraph index. As long as we consider weighted digraphs a straightforward answer is not possible, because there are still as many kinetic parameters as edges in the network which of course influence the dynamics. Definitely, the matrix entries (i.e. edge weights) impose lower and upper bounds on the index [18], [22],
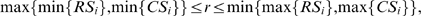
(5)where 
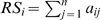
 and 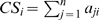 are the 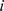
th row and column sums, respectively, and 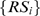 and 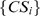 (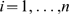) denote the corresponding sets.

A critical result can be derived if the nodes are permuted such that the adjacency matrix is brought into the so-called irreducible normal form denoted 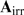 [22]. Then the square matrices aligned in the diagonal 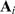 with 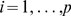, 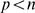 are either irreducible or 1-by-1 zero matrices. By permutation similarity the eigenvalues of 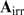 are equal to those of 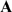 and hence for the spectrum 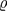 the following holds true (see also [23]):
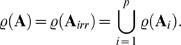
(6)


In fact, the irreducible matrices are the adjacency matrices of the SCCs whereas the acyclic subdigraph of 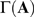 has only zeros in the diagonal of 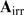. Hence, 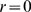 for acyclic graphs in general, as is well-known in graph theory [18], [20]. According to (4) a zero index means that acyclic networks always have a stable off-state irrespective of the values of the activation constants. Thus we focus in the following on the influence of the SCCs.

We will call the set whose elements are the SCCs the *feedback family* of the network and denote their indices by 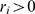. The union of spectra (6) shows that the index of the whole network is determined by the component with maximal index [23],
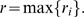
(7)The SCC with maximal index is called the *dominant component* of the feedback family. Relation (7) is intriguing because it implicates that the positive feedback cycles outside of the dominant component are neutral with respect to off-state stability. If the 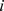-th SCC dominates the network index, condition (4) reduces to 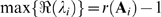. If all or more SCCs have the same maximal index, these components are *dominant representatives* of an index class called the *dominant class* of the network. The index of any network equals the index of its dominant class. Note, that the number of representatives in a dominant class is irrelevant for stability. Unlike the assumption raised by means of numerical calculations [16], this analytical result shows that there is no straightforward relation between number of feedback cycles and off-state stability. Thus, dominance over off-state stability is not an intrinsic property of a single strongly connected motif but rather depends on the context given by the specific composition of the feedback family.

### Kinetic Off-State Robustness and Its Relation to Strongly Connected Motifs

Because 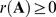 always holds true, condition (4) can provide a natural definition of the network's off-state robustness. The basic idea is that the extent of the parameter space, for which a key function of a system is maintained, can be seen as a possible measure for robustness of a biochemical network towards this function [4], [8].

In the following we confine the treatment to simple (i.e. unweighted) digraphs by setting 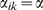 and assume that there is no trans-autoactivation. The resulting binary adjacency matrix is denoted 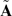 and its index has no values between zero and one [22]. The advantage of restricting the analysis to simple loop-less digraphs lies in the fact that in-degree and out-degree of each node directly reflect the number of upstream activators and downstream targets of a signaling compound, respectively. For an accurate kinetic analysis these restrictions are too strong. However, because our focus is on constraints imposed solely by topology the simplification is reasonable. In the more general case different edge weights could partially compensate each other to allow for stability.

Relation (4) now reads 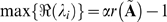 and the critical value for the bifurcation parameter 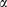 results in 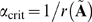. Thus the structural design of a signaling system encoded in the index 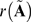 distinguishes parameter regions with the desirable property of stability (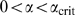) and a region in which a dysfunction of the system by autoactivation cannot be avoided (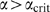). Apparently, the larger the stable regime the more reliably the signaling system performs its function in the sense that additional combinations of the parameters 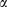, 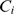 and 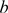 are buffered by the network structure. Therefore, the value 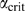 provides a measure for what we call the *kinetic off-state robustness*

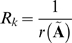
(8)of a network design. This of course results in 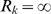 for acyclic networks. In the following we discuss networks with non-empty feedback family. It is appropriate to classify the SCCs as either regular or irregular digraphs because the structural characteristics decisive in kinetic off-state robustness differ in both cases.

#### The Degree of Regular SCCs

A *regular* digraph of degree 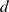 (or 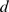-regular digraph), is a digraph where every node has the same in- and out-degree 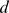. A cycle of arbitrary length thus is classified as a 1-regular digraph. Note, that a higher degree implicates more cycles in regular digraphs. Inclusion relation (5) implies that 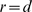 for 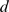-regular components [18]. Thus, the degree of regular SCCs is decisive in kinetic robustness, 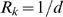. The minimal degree possible, realized in a cycle, confers the highest possible robustness value, 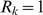. Note, that this equation holds regardless of the length of the positive feedback. The lowest robustness value, 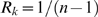, for a network of a given order 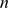 is due to the fact that all networks are spanning subdigraphs of the completely connected simple loop-less digraph, which is regular of degree 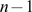 [18]. This implies that regardless of the actual size and topology the off-state is always stable whenever 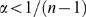 is fulfilled.

#### The Girth and Relative Size of Irregular SCCs

Strong digraphs which are not regular always comprise interlocked cycles of different length. An example for a signaling system with interlocked cycles is the neuronal EGF-pathway studied in [24], exhibiting a SCC with positive feedbacks of length 6 and 7. Whereas the number of non-overlapping cycles does not influence the index, interlocked cycles have a further destabilizing effect and lead to indices 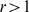. Networks with irregular SCCs are thus always less robust than networks with only cycles in the feedback family.

The specific effect of cycles on the index has been clarified by Brualdi [25] who noticed that cycle structure constrains the index according to 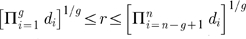. Here, 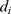 denotes elements from either the in-degree or the out-degree set in an increasing order. The relation shows that along with the number of interactions the *girth*, 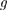, defined as the length of the shortest cycle in the SCC turns out to be an important topological characteristic. In our terms, the SCC with the shortest girth and the greatest number of interactions will most probably be the dominant component. The difference between size and order of the component further constrains the index, since, according to [26], 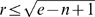 holds.

In summary, if there is evolution towards off-state robustness, cycles should be prevented (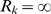). Beyond that, nothing can be said about structural properties in acyclic networks regarding kinetic off-state robustness. If for some reason, for example to achieve a robust bistable response, positive feedbacks are necessary, they should preferably not be interlocked, i.e. the feedback family should comprise only 1-regular SCCs (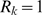). Again, nothing can be said about the number and length of the cycles. However, in the case of interlocked feedbacks further constraints may become relevant. The dominant component should have a small index which may be achieved by keeping the difference between size and order of the SCC small. Since each edge in a SCC is part of a cycle, this also applies to the number of interlocked feedbacks. Moreover, the SCC should be organized such that the girth is as large as possible.

### The Impact of Structural Perturbations - Structural Off-State Robustness

Natural variation among individual cells in a population is a prerequisite of evolution. Thus, within our framework selection pressure towards off-state robustness in signaling systems can only act as long as there are topologies with distinct indices. However, as the spectrum does not fully determine topology there is considerable structural plasticity within index classes, for example acyclic networks may be in-trees or out-trees. To evaluate the significance of those structural properties which vary among networks with the same index, we further elaborate the concept of off-state robustness.

So far, we analyzed the implications of different topologies while assuming the topology of a particular system to be fixed. In the following, we consider the fact that living cells are steadily subjected to variation even on the structural level of signaling systems due to random genetic events, diseases or other exogenous factors. If to each network topology a dynamical system is attached, structural perturbations may affect dynamic properties like off-state robustness. We assume that during evolution signaling networks have been molded into topological designs which support kinetic off-state robustness despite structural defects.

#### A measure for structural off-state robustness

In analogy to the concept introduced in [27] for metabolic networks, we define a *neighborhood* 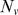 of a particular network structure 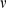 by considering specific structural perturbations, e. g. addition or deletion of a single edge. All neighboring networks 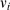 possess an index 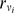. We pursue the following hypothesis: natural selection not only favors networks with a low index but networks having a neighborhood comprised of networks with low indices. Therefore, we set out to determine those design features which support a robust neighborhood to see if they are found in real signaling systems.

In order to assign a robustness value to the neighborhood first we make the simple assumption that all possible perturbations in a certain perturbation mode (see below) are equiprobable. In case of an event the transition probability to any neighbor is 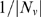, where 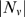 denotes the number of neighbors. Second, for evaluating the index of the neighborhood only those neighbors are taken into account which to some extent still perform the original network function, that is those having similar input-output relations, which is tested by checking reachability between input and output nodes. These neighbors are said to belong to the *functional neighborhood* 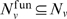 with the index defined as
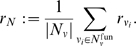
(9)With the analytical results from the first part at hand we can quantify the *structural off-state robustness* of the original network design in analogy to the kinetic robustness, Eq. (8), as 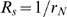.

A network cannot cope with all types of perturbation, and also from the computational point of view it is feasible to confine the analysis to small perturbations which presumably occur more frequently. Here, changes of network order are not considered. The addition of a new signaling protein caused by gene duplication for example is a rare and long-term process [28]. Thus, selection pressure towards structural robustness is assumed to be dominated by perturbations changing only the interaction pattern in the network.

Due to the modular assembly of eukaryotic signaling proteins it is possible that not all interactions of a protein are affected at once. Basically, binding to other proteins and catalytic domains are structurally separated [29], [30]. Based on this knowledge, two different modes of perturbations are analyzed which lead to different compositions of a network's neighborhood.

#### Perturbation mode (a)

Only loss or gain of one interaction is considered, i.e the active site is assumed to be unaffected.

#### Perturbation mode (b)

Additionally, perturbations of the catalytic site are taken into account: Thus, a species 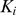 either looses all its outgoing edges or it can loose its specificity which can result from oncogenic mutations as has been reported e.g. in [31], [32].

**Figure 1:**
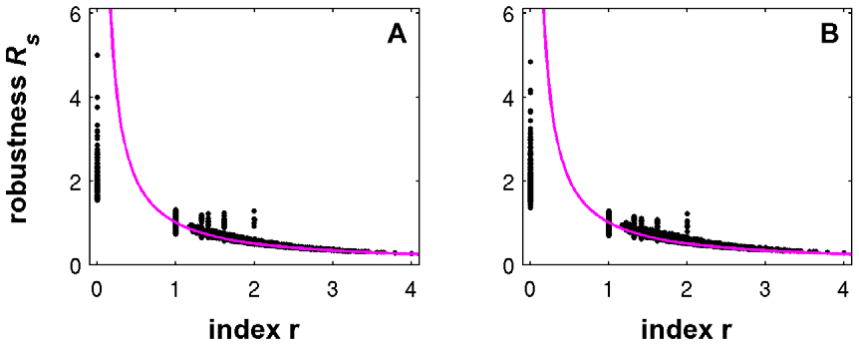
Dependence of structural off-state robustness on network index. Each dot corresponds to one of the 9364 non-isomorphic topologies of order 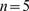. The structural robustness values are calculated for perturbation mode a (left) and b (right). For comparison, the colored curve shows the kinetic off-state robustness, Eq. (8).

First, numerical analysis of digraphs of different order suggests a significant negative correlation between a network's index and its structural off-state robustness for both perturbation modes. That is, a lower kinetic robustness results in a decreased structural robustness. This is illustrated in Figure 1 for all 9364 non-isomorphic digraph topologies of order 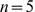. A dot represents a network 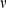 characterized by a certain combination 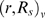. Because acyclic topologies have 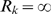 but always possess neighbors with cycles, their structural robustness is finite. For indices 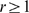, the values of 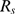 scatter more or less around 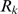, however, for 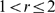 it seems that the functional neighborhood tends to be more robust than the original network. Similar pictures are obtained for 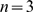 (13 topologies), 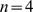 (199 topologies) and 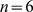 (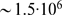 topologies, data not shown).

Despite its correlation with the index it is interesting to see that the structural off-state robustness can vary considerably within the same index class. At least for the acyclic (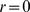) and 1-regular (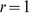) classes the set of networks clearly fans out. Can we find structural properties among index equivalent networks which can explain this diversity? To this end, we chose the index classes 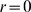 and 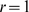 and analyzed all digraphs of order 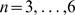. The index classes with 5,942 and 30363 topologies, respectively, were evaluated separately to not introduce some bias. Three candidate features are tested for correlation with structural off-state robustness: connectivity, divergence and path sum.

#### Structural robustness and connectivity

**Figure 2:**
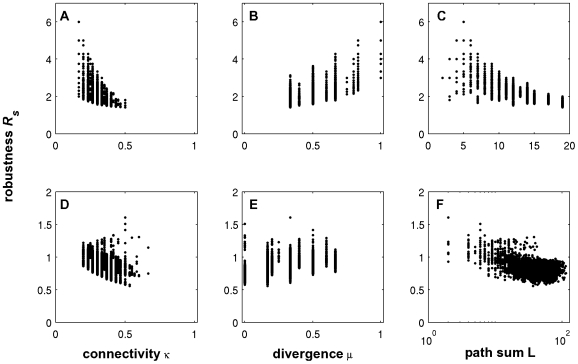
Correlations of topological properties with structural off-state robustness in two index classes. A, B and C show the dependence of acyclic digraphs on connectivity, divergence and path sum, respectively. D, E, and F (log-scale) depict the same for digraphs with only 1-regular SCCs. All non-isomorphic digraphs of order 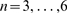 were analyzed using perturbation mode a for the calculation of 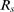.

The influence of connectivity on structural off-state robustness is depicted in Figure 2A for acyclic topologies and Figure 2D for 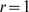. A negative correlation for acyclic networks indicates that maintaining off-state robustness tends to be supported by a low connectivity. This property is reasonable at least from one perspective. A 2-cycle can always be generated by adding an existing edge in reverse direction; therefore, the more edges in the network, the more neighbors exist with 2-cycles. However, there are many networks with identical connectivities but quite different 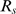-values. Moreover, the robustness of topologies with cycles seems to correlate with connectivity rather weakly.

#### Structural robustness and divergence

The number of sinks and sources, 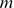, reflects the extent of a network's communication with the environment. To compare networks of different order we introduce the *divergence* 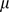 given by 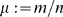, which generally varies in the range of 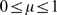. Figure 2B shows that divergence and structural robustness are positively correlated for acyclic networks. The robustness supporting effect of the divergence is due to the fact that sinks and sources cannot be part of cycles. The more sources and sinks that exist in the original network, the less likely the generation of a cycle by an edge addition. However, like in the case of connectivity there are still many networks which do not obey this tendency. Again, no correlation can be observed in the class 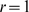 (Figure 2E).

#### Structural robustness and path sum

Generally, the generation of an 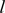-cycle by an edge addition requires the existence of a path of length 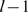 in the original network. Therefore, longer paths and more paths in a network suggest an increased number of neighbors with cycles and thus lower robustness. If there exist 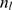 paths of length 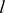 in the network, the *path sum* in the network is defined as
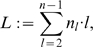
where all paths of length 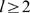 are counted. In case of the path sum both acyclic networks and networks with cycle motifs show a marked tendency of robustness decay with more paths in the network. The calculations suggest that a low path sum is a structural feature which is of importance to maintain off-state robustness against structural perturbations.

### Robustness of Real Signaling Networks

For illustration and to put our hypothesis to the test five signaling systems have been evaluated: The Transpath [33] kinase network which was already analyzed in [17] for other properties and some recent canonical signaling networks [34]–[37] retrieved from the Science STKE database [38]. The positive interaction maps of the STKE networks were derived as follows. First, the negative interactions and those whose sign is currently unknown were deleted. Any node becoming isolated by this procedure was ignored. Second, because we are considering only the signal-off status, all nodes defined as an external signal are discarded. Because the corresponding edges have to be deleted as well, again some nodes may become isolated. The topological properties shown to be important for our study are summarized in Table 1.

**Table 1:** Topological characteristics relevant for ground state robustness in real networks.

Network	Nodes *n*	Edges *e*	Index *r* (*R_k_*)	Rob. *R_s_*	Conn. *κ*	Div. *μ*	Path Sum *L*
**Transpath**	86	171	0 (∞)	6.794	0.023	0.605	635
**Adrenergic**	35	42	0 (∞)	7.579	0.035	0.276	174
**B Cell Antigen**	29	37	0 (∞)	3.878	0.046	0.343	213
**TGF** ***β***	16	33	0 (∞)	3.735	0.138	0.250	36
**IFN** **γ**	6	8	0 (∞)	1.851	0.266	0.333	15

Each network is characterized according to the topological aspects deemed significant according to the results part. Networks 2–5 are derived from the STKE database as described in the text.

We want to demonstrate that there exist real signaling systems that may indeed exhibit off-state robustness and that the corresponding topological properties are not the product of chance. For this, structural features are compared with random counterparts to assess their statistical significance. Random networks can be created by different means. Here a rewiring method is used which is described in [39]. Starting with the real network, repeatedly, two edges are chosen at random, their target nodes or their nodes of origin are exchanged, provided the resulting edges do not already exist. This procedure ensures that in the rewired network order, size and the in-degrees as well as out-degrees for each node are preserved. As a consequence, of the three properties considered above, only the path sum differs between the random networks.

**Figure 3:**
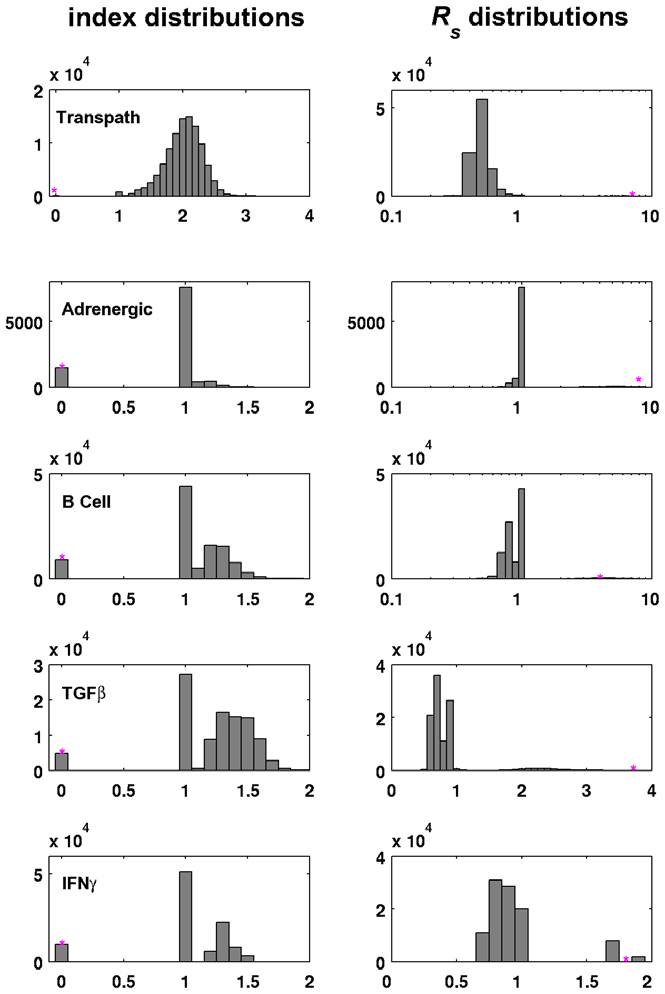
Histograms of dynamic robustness in randomized signaling networks retrieved from databases. For the Adrenergic pathway 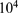 random networks were evaluated, for all other signaling systems 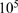. The distribution of the indices of the randomized networks are depicted in the left column. The right column shows the structural robustness values for the perturbed networks. The asterisks mark the values for the original networks. Perturbation mode a has been used to calculate 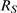.

The results for the five biological networks are depicted in Figure 3. The graphs in the left column depict the distributions of the indices of the perturbed networks, and the graphs in the right column represent the distributions of their structural robustness values. All five networks are acyclic and the index distributions clearly indicate that the maximal kinetic off-state robustnesses can not be expected by chance. For the Transpath network for example, only 35 out of 100,000 random networks are acyclic. Therefore, an acyclic network design is most likely a striking feature. Further, the structural robustness for three of the five networks is the highest observed for all perturbations. Only for the B Cell Activation network and for the IFN*γ* pathway, perturbed network structures with a slightly higher structural robustness were found. We hypothesize that these findings also reflect evolution towards structural off-state robustness. Table 2 gives an overview of the p-values of all signaling networks analyzed. All analyzed networks have a significantly low index and high structural off-state robustness. The path sums are all lower in the original networks than in the random samples, however, compared to the values of random networks being in the same index class only the TGF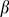 and (weakly) the IFN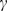 pathways seem to have significantly low p-values. To assess the effect of connectivity and divergence the original networks would have to be tested against random networks generated by different means, for example by generating Erdös-Renyi graphs of the same order.

**Table 2:** Statistical significance (p-values) of topological characteristics in real networks tested against randomly rewired samples.

Network	Index *r*	Robustness *R_s_*	Path Sum *L*
**Transpath**	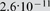	≈0*	0.2232
**Adrenergic**	0.0445	0.0015	0.2413
**B Cell Antigen**	0.0117	0.0274	0.7920
**TGF** ***β***	0.0017	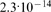	0.0031
**IFN** **γ**	0.0178	0.0033	0.0795

* Properties which can differ between the original network and the random samples of 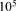 rewired networks, respectively, have been tested for significance by calculating the p-value from a normal distribution using the z-score. The original path sum has been compared to the path sums of random counterparts being in the same index class as the original network.

The p-value for the structural robustness in the Transpath kinase network is extremely low, the corresponding p-value for the neighborhood index, 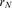, is 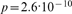.

## Discussion

Kinetic parameters of biochemical systems can be altered by mutations resulting in perturbed activities and are subject to fluctuations due to variations in the environment (pH, temperature, ionic strength). These sources of reaction rate noise may have a profound effect on the dynamic behavior of a system. Moreover, in many cases the same topology is realized with different proteins in different organisms and cells (orthologs and paralogs) [40]. These supposedly have different kinetic properties. A sensitive signaling design could not cope with such a diversity.

In this paper we argue that the design of signaling systems should have evolved to support the maintenance of functionality as reflected by a stable ground state. This frees up the kinetic parameters of the system to meet other demands. Therefore, both a higher kinetic and structural off-state robustness of network design could confer a better evolvability [41], [42].

With reference to the robustness principle, our approach is somewhat complementary to that of Shinar et al. [43]. There, the signal-on case is considered in which the output should accurately match the input despite variations in components. However, through our systematic approach we could analyze a particular set of models and highlight topological features which to our knowledge have not been considered before in the context of signaling networks. The generality of our approach required us to make some drastically simplifying assumptions, such as equal rate constants for deactivation processes. Clearly, for many biological systems such simplifications are unrealistic. To overcome the simplifications made on the rate constants, a future prospect could be to combine our notion of robustness with that introduced in [13] to also quantify the parameter ranges of stable behavior for cases for which the analytical results presented here do not hold. Another limitation of our theoretical framework is that we did only analyze positive regulatory structures and it would be an interesting and obvious extension of our approach to also include the stabilizing or destabilizing effects of negative feedback loops.

Nevertheless, our studies provide insight into how graph properties of the underlying networks influence their robustness, both with respect to dynamic and structural perturbations. Indeed, the apparent lack of cycles and a higher compactness, which is related to a lower path sum, has also been noted by other authors [44]. These observations can in fact be explained in the light of ground state robustness. For selected real signaling networks, we have shown that these and other topological features are characteristic for evolved systems and are not expected to have appeared by chance. Our results demonstrate that, despite the simplifying assumptions, conclusions with a general validity may be drawn and support the view that ground state robustness is a systemic constraint which puts selection pressure on signaling network topologies during evolution.
